# Shoal size as a key determinant of vulnerability to capture under a simulated fishery scenario

**DOI:** 10.1002/ece3.4107

**Published:** 2018-06-11

**Authors:** Davide Thambithurai, Jack Hollins, Travis Van Leeuwen, Anita Rácz, Jan Lindström, Kevin Parsons, Shaun S. Killen

**Affiliations:** ^1^ Institute of Biodiversity, Animal Health and Comparative Medicine University of Glasgow Glasgow UK; ^2^ Cape Eleuthera Institute Eleuthera The Bahamas

**Keywords:** collective behavior, evolutionary trap, fisheries‐induced evolution, metabolic rate, sociality

## Abstract

Group living is widespread among animals and has a range of positive effects on individual foraging and predator avoidance. For fishes, capture by humans constitutes a major source of mortality, and the ecological effects of group living could carry‐over to harvest scenarios if fish are more likely to interact with fishing gears when in social groups. Furthermore, individual metabolic rate can affect both foraging requirements and social behaviors, and could, therefore, have an additional influence on which fish are most vulnerable to capture by fishing. Here, we studied whether social environment (i.e., social group size) and metabolic rate exert independent or interactive effects on the vulnerability of wild zebrafish (*Danio rerio*) to capture by a baited passive trap gear. Using video analysis, we observed the tendency for individual fish to enter a deployed trap when in different shoal sizes. Fish in larger groups were more vulnerable to capture than fish tested individually or at smaller group sizes. Specifically, focal fish in larger groups entered traps sooner, spent more total time within the trap, and were more likely to re‐enter the trap after an escape. Contrary to expectations, there was evidence that fish with a higher SMR took longer to enter traps, possibly due to a reduced tendency to follow groupmates or attraction to conspecifics already within the trap. Overall, however, social influences appeared to largely overwhelm any link between vulnerability and metabolic rate. The results suggest that group behavior, which in a natural predation setting is beneficial for avoiding predators, could be maladaptive under a trap harvest scenario and be an important mediator of which traits are under harvest associated selection.

## 1 INTRODUCTION

Group living can provide wild animals with significant benefits associated with predator avoidance and foraging (Pitcher, Magurran, & Winfield, 1982). Animals in groups can reduce their risk of predation through a variety of mechanisms, including increased vigilance and a dilution of risk for individual groupmates (Krause & Ruxton, 2002). Groups of animals also tend to find food patches more consistently and can share information regarding potential food sources (Ekman & Hake, 1988; Giraldeau & Beauchamp, 1999). The anti‐predator and foraging benefits of group living can also interact as individuals in groups are generally more active and spend more time foraging instead of being vigilant for predators (Krause & Ruxton, 2002).

Given the extreme importance of group living for foraging and predator avoidance, it seems reasonable that social dynamics may also influence the degree, to which individuals are vulnerable to capture by humans under anthropogenic harvest scenarios such as hunting or fishing. Humans are extremely effective predators, often exerting higher mortality on animal populations than natural predators (Darimont, Fox, Bryan, & Reimchen, 2015), with potential evolutionary effects on wild populations (Haldane, 1942; Hutchinson, van Oosterhout, Rogers, & Carvalho, 2003; Jachmann, Berry, & Imae, 1995; Macnair, 1987; Voipio, 1950). Of particular concern in this context are fish populations, as they are under very high levels of exploitation with life histories that typically involve group living (Jørgensen et al., 2007). Brown and Warburton (1999) found that rainbowfish *Melanotaenia duboulayi* in larger groups were more successful at escaping capture by trawls, possibly due to the additional information that shoal mates provided during escapes. To date, however, there is little knowledge about how group living affects vulnerability to passive capture methods, such as trapping, which rely on individuals to encounter and voluntarily interact with the deployed gear. Increased rates of activity may increase encounters with deployed traps, and individuals may follow groupmates into traps while foraging, resulting in increased susceptibility to capture when fish are in larger groups.

Individual predation risk can also be related to physiological traits. For example, in some contexts, levels of spontaneous activity and risk‐taking while foraging can be positively linked with an animals' metabolic rate (standard metabolic rate in ectotherms, SMR, the baseline level of energy intake needed to sustain life) (Careau & Garland, 2012; Killen, Marras, Ryan, Domenici, & McKenzie, 2012; Metcalfe, Van Leeuwen, & Killen, 2015). Furthermore, individuals with a higher metabolic rate also tend to be less social, presumably to reduce competition for food items with potential groupmates. These links between individual metabolic rate and behavior may also be highly relevant for determining which individual fish are most vulnerable to capture in fishing scenarios (Alós, Palmer, Rosselló, & Arlinghaus, 2016; Diaz Pauli & Sih, 2017; Hollins et al., 2018; Kern, Robinson, Gass, Godwin, & Langerhans, 2016; Killen, Nati, & Suski, 2015). For instance, individuals with a higher metabolic rate may be more likely to encounter traps if they spend more time searching for food or be more willing to enter a discovered trap if they are bolder or more attracted to bait (Hollins et al., 2018). Within and across species, SMR can also be functionally related to the maximum metabolic rate achievable by an animal (MMR), due to increased maintenance costs of increased mitochondrial density, muscle mass, and cardiovascular machinery even when the individual is at rest (Auer, Killen, & Rezende, 2017; Killen, Glazier, et al., 2016). Therefore, even though passive gears are stationary and do not elicit intense exercise during capture, it is plausible that traits such as MMR could also be under correlated selection by such gear. An important consideration, however, is that individuals with a higher SMR tend to be less social (Killen, Fu, Wu, Wang, & Fu, 2016), probably due to costs associated with resource‐sharing in groups. It is therefore entirely possible that individuals with an increased energy demand may be less likely to follow conspecifics into passive fishing gears and have reduced vulnerability to capture while in groups. Social effects on vulnerability to capture may also outweigh or obscure any potential vulnerability to capture related to metabolic traits, and thus perhaps dilute any potential for selective effects on these characteristics.

Passive gears, such as pot traps, which consist of a funnelled entrance which facilitates fish entry but makes escape difficult, are increasingly considered for some fish species as an alternative to trawling due to their reduced potential for damage to benthic communities, more sustainable exploitation patterns, reduced discards, and the ability to return bycatch relatively unscathed (Jennings & Kaiser, 1998). Unfortunately, however, we still know little about the factors that determine individual vulnerability to capture by trapping in fish and the potential for selective effects. To examine these issues, we conducted small‐scale simulations of a trap fishery targeting individuals in different ‐group sizes shoals. We also measured the metabolic traits of all focal fish using intermittent‐flow respirometry. Small‐scale fishing simulations are a key tool for understanding the mechanisms underpinning vulnerability to capture and the effect of environmental variables on capture success (Brown & Warburton, 1999; Clark, Messmer, Tobin, Hoey, & Pratchett, 2017; Diaz Pauli, Wiech, Heino, & Utne‐Palm, 2015; Killen et al., 2015). As a starting point for understanding the effects of social behavior and metabolic demand on vulnerability to passive gears, we focus on the key phase of the fishing sequence where fish are in the general proximity of a deployed gear, but must precisely locate and voluntarily interact and enter the trap. Indeed, recent work has shown that while encounter with gears within a broad habitat is a requirement for capture success, it is smaller‐scale interactions between fish and gear that are likely more important determinants of which individuals are ultimately captured (Monk & Arlinghaus, 2017). We used wild zebrafish *Danio rerio*, a small cyprinid native to southeast Asia, to answer two main questions: (1) does shoal size affect the vulnerability of individual fish to trap capture?; and (2) does shoal size modulate any potential links between metabolic rate and vulnerability to capture by trap among individual fish? We hypothesized that vulnerability to harvest would increase with larger shoal size and that fish with higher metabolic demands would be most vulnerable.

## 2 METHODS

### 2.1 Study organisms

Wild zebrafish were obtained by dip nets from the Kosi river, India (source 26°54′47″N 87°09′25″E). Fish were shipped to the University of Glasgow (Glasgow, Scotland, UK) and maintained in several 300‐L stock tanks (120 long × 61 wide × 47 high cm) at equal densities before testing. These tanks were supplied with recirculating, ultraviolet‐treated water maintained at 28°C on a 13:11 hr light:dark cycle and enriched with plastic plants and sand. Zebrafish were fed ad libitum daily on a combination of commercial feed and live *Artemia nauplii*. Fish were held in the laboratory under these conditions for approximately 4 months prior to the start of the study.

### 2.2 Measuring vulnerability to capture

Three weeks prior to the start of behavioral trials all individuals (*n* = 159) were tagged using visual implant elastomer (VIE) (Northwest Marine Technology, WA, USA) in one of four dorsal tag locations. Wet mass and fork length were also measured and recoded for each individual (mean fork length = 32.5 ± 2.47 mm, mean wet body mass = 0.39 ± 0.10 g). These fish made up the group of focal individuals and were housed separately from their initial stock populations at a density of <6 fish per litre in a zebrafish rack system (Z‐Hab system, MBK Ltd, Nottingham, UK) but under the same temperature and light conditions as above. Four treatments were tested during the experiment: focal fish were tested individually (IND; *n* = 41) or with the addition of two (IND_+2_; *n* = 40), four (IND_+4_; *n* = 40) or six (IND_+6_; *n* = 38) shoal mates, with each fish only experiencing a single trial. Shoal fish continued to be housed in their initial stock tanks and taken daily as needed.

**Figure 1:**
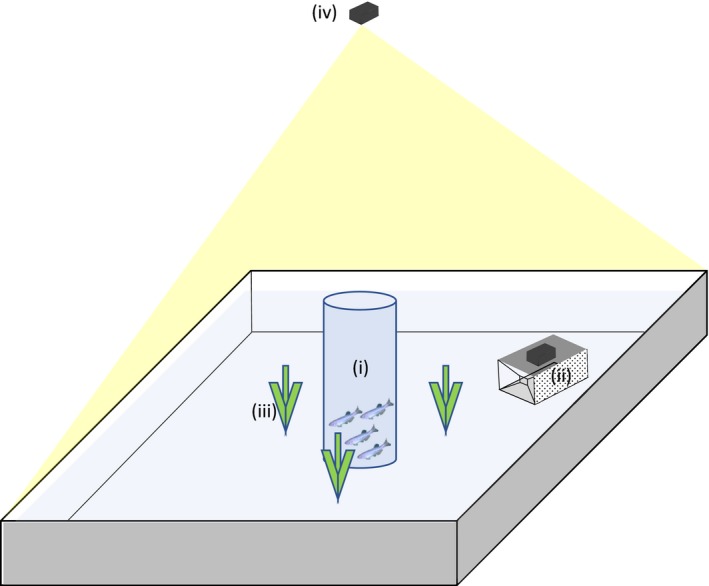
Experimental set‐up used in the trapping vulnerability tests. Here, (i) is the acclimation tube, (ii) is the trap with mounted camera, (iii) denotes the plants, and (iv) is the overhead camera

In shoaling treatments, the behavior of one focal individual was quantified according to the procedures described below. Each trial was performed with a different focal fish and all fish were naïve at the start of the experiment. Fish were tested for their vulnerability to trapping in a behavioral arena (76 long × 56 wide × 21 high cm) supplied with recirculating carbon‐filtered water (AVEX 1000) to a depth of 9 cm and temperature controlled to 28.0 ± 0.5°C. Filtration and temperature control occurred in a separate reservoir, and water was fed and removed from the arena through a looped system using pumps. Three artificial plants were placed in the arena to encourage exploration and reduce stress, as well as a glass cylinder which served as an acclimation area (Figure 1). The entire experimental setup was housed within a frame covered with opaque curtains to minimize disturbance to the fish during trials. Lighting during the experiment was provided by four 8 W daylight lamps mounted at each corner of the arena.

A custom‐made scaled replica finfish trap measuring 135 × 80 × 73 mm baited with commercial fish pellets (6 mm Goldfish pellets, Vitalis Aquatic Nutrition, Doncaster, UK) was used to simulate capture. The trap consisted of a metal frame covered in white netting (<1.5 mm mesh size) with two inverted funnel entrances measuring 3 cm^2^ located at each end of the trap. The top of the trap was constructed from clear 3 mm Plexiglas to allow mounting of a GoPro Hero 4 (16:9 Full HD, 720p; GoPro, San Mateo, California, USA) camera used to monitor the movement of fish in and out of the trap.

Focal and stimulus fish were fasted for 36 hr prior to the commencement of trapping trials and were isolated from the other fish in their common holding tanks during this time, both to minimize stress during testing and to standardize hunger and attraction to the baited trap. The order of treatments across trials was randomized. At the beginning of a trial, focal fish along with any shoal mates were placed in the clear glass cylinder in the center of the arena for five minutes. Following this acclimation period, the cylinder was remotely lifted through a pulley system and fish were allowed to explore the arena and monitored for 20 min. Fish were observed via an overhead camera (Logitech HD Webcam c920; Logitech Europe S.A., Lausanne, Switzerland) mounted directly above the trap. Trap location was randomized across four predetermined areas (each corner of the arena with the trap being >5 cm away from the walls of the arena) taking care not to obstruct entrances.

An observer monitored the video in real time and noted the time of first entry of the focal fish to the trap in seconds (*T*_e_). In some cases, fish exited and entered the trap multiple times, and so the total number of entrances made by focal fish (*N*_e_) was also quantified. The total time spent within the trap (*T*_t_) by the focal fish was also calculated using data from the intervals between any trap exits and re‐entries. At the end of each trial all fish, whether captured or not, were removed from the arena and returned to holding tanks. Focal fish were kept separate from shoal fish to allow subsequent measurement of oxygen uptake. To maintain water quality, a 10% water change was conducted between trials, with all water within the behavioral arena being changed at the end of each trial day.

### 2.3 Estimates of metabolic traits

After trapping trials, all focal fish were then measured for oxygen uptake using intermittent‐flow respirometry to estimate SMR and MMR (at least 10 days following trapping trials; mean = 21 days; range = 10–32 days). Fish were haphazardly removed from their holding tanks using dip nets and metabolic rate was estimated as the rate of oxygen uptake using intermittent‐flow respirometry. Maximum metabolic rate (MMR) was measured after exhaustive exercise in a 30 L swim tunnel (Loligo Systems, Tjele, Denmark). This method assumes that maximum rates of oxygen uptake are achieved during the recovery from the bout of exhaustive anaerobic exercise (Killen, Norin, & Halsey, 2017). Fish were initially exercised within the swim tunnel at 6 cm/s and allowed to orientate and acclimate for 1 min. Speed was then gradually increased to 50 cm/s—the approximate critical swimming speed of zebrafish—and was used to induce anaerobic swimming (Palstra et al., 2010). The fish were then observed at this speed until they tired and contacted the back of the flume chamber. Following contact with the back of the flume, speed was lowered to <1 body length per second and the fish was mechanically stimulated to swim using a dip net. After the third contact with the flume chamber, the fish was removed and transferred into an individual cylindrical 58 ml glass respirometry chamber connected to an intermittent stopped‐flow respirometry system. Time between fish exhaustion (mean = 133 ± 83 s) and transfer to the respirometry chamber was always <60 s.

Within the respirometry chambers, water oxygen content was quantified once every 2 s using a Firesting 4‐channel oxygen meter and associated sensors (PyroScience GmbH, Aachen, Germany). Respirometers were placed within an aerated, rectangular, temperature‐regulated water bath (28.4 ± 0.09°C; 50 L), and were shielded from disturbance and direct lighting by an opaque plastic blind. Water mixing within each respirometer was achieved with a peristaltic pump that moved water through the chamber and around an external circuit of gas‐impermeable tubing. Every 10 min, an automated flush pump would switch on for 2 min to flush chambers with fresh water, and, when switched off, sealed the respirometers to allow the decrease in oxygen concentration to be measured. To estimate MMR, we calculated rates of oxygen uptake for each 2 min time interval throughout the first 20 min of recovery immediately following the exhaustive exercise described above; MMR (mg O_2_ per hour) was taken as the highest rate of aerobic metabolism during this period. After measurement of MMR, fish remained in the same respirometry chambers overnight to allow for the estimation of SMR. Individuals were then removed from the respirometer at around 09.00 the following day. They were then immediately measured for wet mass and standard length. Whole animal SMR (mg O_2_ per hour) was estimated as the lowest 10th percentile of measurements taken throughout the measurement period. The first 5 hr of confinement as well as the last 3 hr were discarded for calculation of SMR as the oxygen consumption of the fish was found to be elevated during these periods.

### 2.4 Statistical analyses

Statistical analyses were performed in R 3.2.2 (R Development Core Team). Two general linear models (GLM) with Gaussian distributed error variances were constructed with either *T*_e_ or *T*_t_ (s) as the response variable, shoal size as a categorical explanatory variable, and wet mass, fork length, SMR, and MMR of focal fish as continuous explanatory variables; interaction terms shoal size:SMR and shoal size:MMR were also included. Time of first entry (*T*_e_), wet mass, fork length, SMR, and MMR were log‐transformed to conform to model assumptions of linearity and homoscedasticity (Zuur, Ieno, & Elphick, 2010). All time‐based metrics used were analysed as proportions. In all cases, we present the best fitting models as determined by the Akaike Information Criterion (AIC). Where SMR was kept in the model, log (wet mass) was retained, regardless of AIC, to control for the allometric scaling of metabolic rates. Variance inflation factors were calculated for the explanatory variables included in the model to remove potential collinearity, threshold value for removal was set at 3 following Zuur, Ieno, Walker, Saveliev, and Smith (2009). One‐way ANOVAs were also used to investigate potential differences in mass and length among treatment levels (IND to IND_+6_). Data for *N*_e_ was found to be overdispersed as indicated by the ratio of residual deviance to degrees of freedom in initial model runs, thus a third model was constructed using a negative binomial distribution and fitted using the function *glm.nb* from the *Mass* package (Venables & Ripley, 2002). Model structure remained similar to the first GLM, with only *N*_e_ replacing *T*_e_ as response variable. To examine the decay in the number of focal free‐swimming fish in the arena over the duration of each trial predicted trapping rates were estimated using the function “survfit” from the R package survival (PSA; R package “survival” (Therneau, 2015). This allowed the visualization of the theoretical harvest in each group size using Kaplan–Meier curves.

## 3 RESULTS

**Figure 2:**
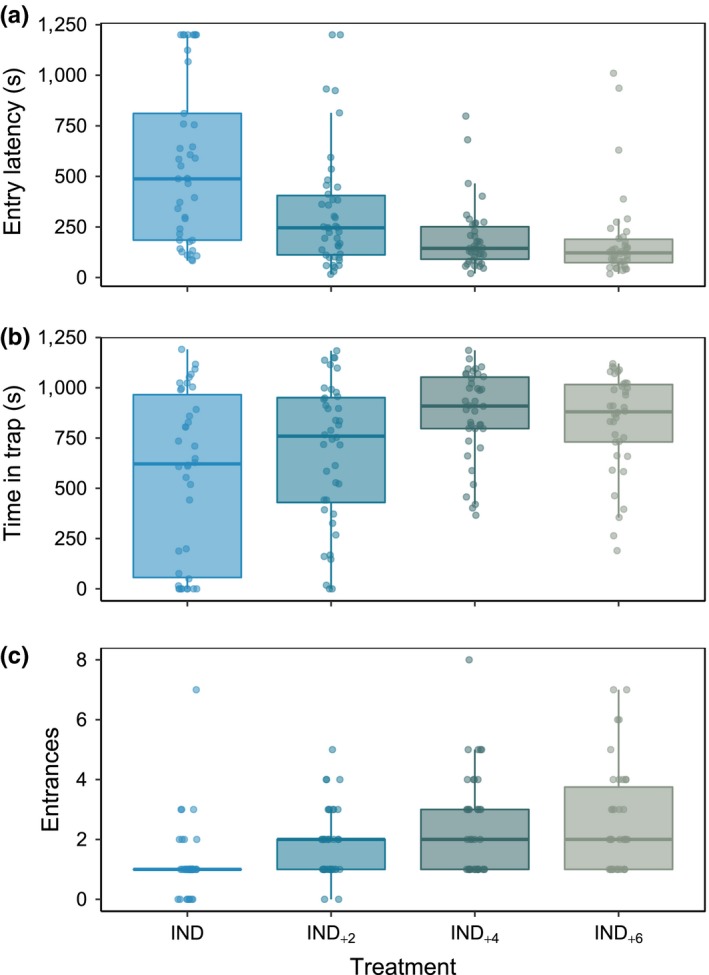
The effects of shoal size on individual vulnerability to capture by trap, as estimated by: (a) time until first trap entry; (b) total time spent within the trap during a 1,200 s deployment; and (c) total number of trap entries. Each data point overlaid on Tukey‐style boxplots is data for one fish. Boxplot lower and upper hinges represent the 25th and 75th percentiles, respectively; the horizontal line within the box represents the median; the length of whiskers represents the range data points between each hinge and 1.5 ×  the difference between the 25th and 75th percentiles. Data beyond these limits are outliers

Total capture rate across trapping trials was 94%; mean *T*_e_ for focal fish among treatments was 323 ± 326 s (mean ± *SD*) while *T*_t_ was 735 ± 340 s. Increasing shoal size caused a significant decrease in *T*_e_ for focal fish and an increase in *T*_t_ (Figure 2, Table 1). Both IND_+6_ and IND_+4_ differed strongly from IND, while focal fish with two conspecifics differed less, especially in terms of time spent in trap. For instance, lone fish, on average, took over three times longer to enter the trap as compared to fish grouped with six conspecifics and spent nearly 197 s less time in the trap (Figure 2).

**Table 1:** Parameter estimates of general linear models assessing factors influencing time until trap entry, total time in trap, and the number of trap entries. SMR = standard metabolic rate. Here IND represents the reference level for the analyses and is included in the Intercept

Model	Term	Estimate	*SE*	*df*	*t*	*p*
Time to entry (*T*_e_)	Intercept	6.046	0.345	138	17.528	<.0001
	IND_+2_	−0.675	0.204		−3.318	.001
	IND_+4_	−1.005	0.206		−4.882	<.0001
	IND_+6_	−1.222	0.208		−5.866	<.0001
	log (SMR)	0.233	0.120		1.941	.054
	log(wet mass)	−0.558	0.310		−1.805	.073
Time in trap (*T*_t_)	Intercept	0.443	0.04	140	9.837	<.0001
	IND_+2_	0.115	0.061		1.880	.062
	IND_+4_	0.283	0.062		4.504	<.0001
	IND_+6_	0.253	0.063		4.002	.0001
**Model**	**Term**	**Estimate**	***SE***	***df***	***z***	***p***
No. entries (*N*_e_)	Intercept	0.084	0.163	140	0.514	.607
	IND_+2_	0.471	0.204		2.307	<.05
	IND_+4_	0.842	0.194		4.322	<.0001
	IND_+6_	0.814	0.196		4.142	<.0001

The mean *N*_e_ among treatments was 1.9 ± 1.5 (mean ± *SD*). *N*_e_ increased when fish were in larger groups, although similarly to *T*_e_, the greatest differences found were between IND and IND_+6_ (Table 1; Figure 2). The mass and length of the focal fish did not differ among treatments (ANOVA: *F*_3,140_ = 1.480, *p* = .22; ANOVA *F*_3,140_ = 0.996, *p* = .397).

**Figure 3:**
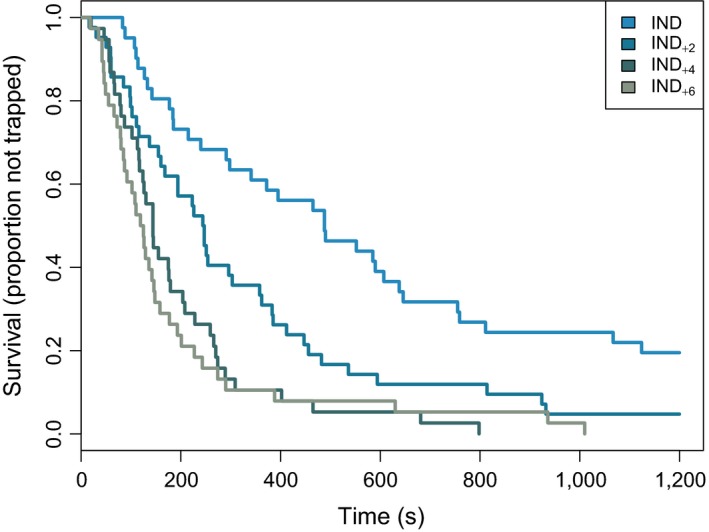
Kaplan–Meier curves of the survivorship of focal zebrafish (*Danio rerio*) trialled at different shoal sizes (IND *n* = 41; IND_+2_ *n* = 40; IND_+4_ *n* = 40; IND_+6_ *n* = 38)

Kaplan–Meier curves built using the *T*_e_ data indicated that the population of IND stabilized with around 20% of fish remaining uncaptured until the end of the trial, while for IND_+2_ approximately 5% of fish remained uncaptured (Figure 3). In contrast, all focal fish in the IND_+4_ and IND_+6_ fish were captured. Over the duration of the trial, a steeper decline in focal fish remaining at large was observed in larger shoals, indicating a higher rate of harvest when compared to smaller shoal treatments. However, the percentage change between IND_+4_ and IND_+6_ was relatively small (<5%).

**Figure 4:**
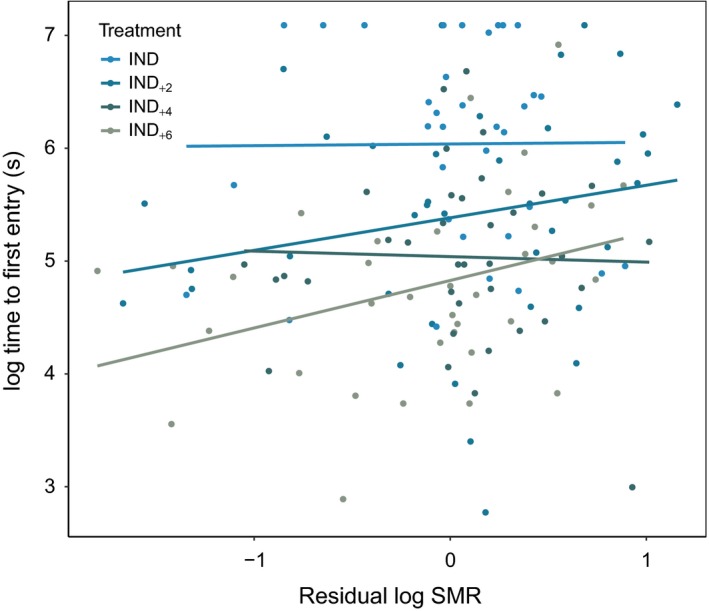
Relationship between residual log standard metabolic rate (mg O_2_ per hour) and time to trap entry (s) at various shoal sizes (IND *n* = 34; IND_+2_ *n* = 37; IND_+4_ *n* = 38; IND_+6_ *n* = 35). Lines represent least squares regression for each treatment (IND 0.01490 * SMR + 6.0377; IND_+2_ 0.27353 * SMR + 5.38353; IND_+4_ 0.06348 * SMR + 5.03955; IND_+6_ 0.40622 * SMR + 4.82809)

There was suggestive evidence that individuals with a higher SMR took longer to enter the traps (i.e., had a greater *T*_e_; Figure 4, Table 1), although there was no evidence that this trend was modulated by shoal size. There was no evidence the SMR was related either *T*_t_ or *N*_e_. Similarly, there was no evidence that MMR was related to either *T*_e_, *T*_t_, or *N*_e_.

## 4 DISCUSSION

Fish in larger groups entered traps much faster and spent more time in the trap as compared to individual fish or those in smaller groups. A major benefit of group living in animals is the reduced risk of predation experienced by individual group members (Krause, Butlin, Peuhkuri, & Pritchard, 2000; Krause & Ruxton, 2002; Pitcher & Parrish, 1993; Pitcher et al., 1982; Stoner & Ottmar, 2004). In contrast, the results of this study suggest group behaviors are exploited and may be maladaptive for fish in the context of trap harvest scenarios. The strong, over‐riding effect of social behavior on vulnerability to capture not only suggests that collective behavior may be related to capture vulnerability, but also that the influence of social group members may attenuate direct selective pressure on other traits within individual animals. Contrary to our expectations, SMR showed little relation with vulnerability to capture and, if anything, it was fish with a higher SMR that took longest to enter traps (Figure 4). These results are of broad interest for understanding the influence of group behavior and metabolism on vulnerability to harvest, as well as other possible pathways for correlated selection on traits (Allendorf & Hard, 2009).

### 4.1 The effect of shoal size on vulnerability to capture

There are several potential explanations for why fish in larger groups entered traps faster. The capture of fish by trap gear can be broken down into several phases: (1) activity before trap discovery; (2) bait detection and localization; (3) trap discovery and entry; and 4) potential escape (He, 2010). At each of these phases, fish in groups may be more prone to capture due to a combination of increased exploration and foraging efficiency, leader‐follower dynamics, and intraspecific competition. Fish in groups, for example, are generally more active than individual fish (Ward, Thomas, Hart, & Krause, 2004) and so likely have an increased probability of encountering a deployed trap. Indeed, with many eyes searching, groups of fish are known to find food patches more consistently (Pitcher et al., 1982). After trap discovery, fish may be more willing to approach the trap (essentially a novel object) when they have the perceived safety provided by the group. Finally, once an individual enters the trap, others will be more likely to follow, although the exact nature of this response may vary among species with differing shoaling dynamics and may also be context specific. In some species, bolder individuals are less social, displaying reduced shoaling, higher levels of exploration and greater reliance on individual information, while shy individuals are known to have stronger shoaling tendencies (Ward et al., 2004); although such relationships can be altered by social context. Fish which would normally display low exploration at an individual level, and therefore low vulnerability to capture, potentially adjust their behavior to that of the more exploratory conspecifics in the group, thereby rendering them more susceptible to capture than they would be otherwise (Dyer, Croft, Morrell, & Krause, 2009). This homogenizing effect of group dynamics could effectively dilute selection on traits at the individual level. This perspective contrasts with the view that passive fishing gears select against bolder personality types, leading to population‐wide shifts toward timid phenotypes (Arlinghaus et al., 2016). More work is needed to understand the extent to which social dynamics override selection on individual behavioral traits in the context of fisheries harvest.

Interestingly, although vulnerability generally increased with shoal size, there was little difference in *T*_e_ or *T*_t_ between the two largest shoal treatments (Figure 2). It is possible that at the largest shoal sizes intraspecific competition becomes a limiting factor, preventing some individuals from entering the trap. Such constraining mechanisms have been witnessed in patch foraging (Robakiewicz & Daigle, 2004). Another possibility is that some level of sensory saturation occurred constraining decision‐making and therefore affecting the rate of capture. Even in large moving shoals fish are known to limit their interactions to a few neighbors in their vicinity (Herbert‐Read et al., 2011; Tien, Levin, & Rubenstein, 2004). In our study, the greater number of individuals in IND_+6_, rapidly moving and offering simultaneous and contrasting information in proximity of the trap possibly led to a plateau in capture efficiency. This is perhaps also true of the overall number of entries made by focal fish, as between the two largest shoal treatments the average *N*_e_ was nearly identical (Figure 2). Overall fish in larger shoals escaped and re‐entered the trap more frequently. Such escape behavior is mirrored in wild fisheries: in fact relatively high escape rates have been reported in trap fisheries with up to 34% of fish exiting traps prior to hauling, and in some cases, longer trap deployment times have been shown to be less effective owing to high escape rates (Cole, Alcock, Tovey, & Handley, 2004). It should be noted that although fish in larger shoal treatments escape more often they are also re‐captured more often, most likely as a result of the higher attraction offered by fish in the trap, which is intrinsically higher in larger treatments.

Shoaling tendency in fish has been shown to be both heritable (Dochtermann, Schwab, & Sih, 2015; Wright, Rimmer, Pritchard, Krause, & Butlin, 2003) and repeatable (Magnhagen & Bunnefeld, 2009; Ward et al., 2004). Therefore, if more social fish tend to be found in larger groups in the wild, then it is possible that sociability as a trait could be under selection in trap fisheries. There may also be more direct effects of sociability on trap vulnerability. For example, it is possible that less social fish within groups of a given size may be less likely to follow conspecifics into a trap. Additional work, in which individual sociability and vulnerability are quantified is needed to resolve these issues, and the extent to which selection on sociability may have evolutionary effects on collective behaviors associated with foraging, energy‐saving during group locomotion (Couzin & Krause, 2003), reducing risk of predation (Ioannou, Guttal, & Couzin, 2012; Landeau & Terborgh, 1986) and migration (De Luca, Mariani, MacKenzie, & Marsili, 2014).

### 4.2 Metabolic rate and vulnerability to capture

We found some evidence that fish with a higher SMR took longer to enter traps than those with a lower SMR. This is contrary to our initial hypothesis that individuals with a higher SMR may be more likely to enter traps if they are more exploratory or are more motivated to consume bait. A possible explanation for this is that fish with a higher SMR may avoid conspecifics that have already entered a trap, especially at higher densities (e.g., when several fish are in the trap) due to increased competition. Indeed, it has been observed that fish with a relatively high SMR are less social, preferring to locate themselves further away from a group of conspecifics (Killen, Fu, et al., 2016). It is possible that individual fish were timid when foraging in isolation, perhaps masking any correlation between intrinsic energetic requirements and capture vulnerability.

Unlike previous work on the relationship between trawling vulnerability and metabolism, we did not find a strong relationship between SMR/MMR and trapping vulnerability. This is perhaps not surprising given that swimming capacity, which is thought of as significant component of selection in trawling, is tightly coupled with whole animal metabolic traits (Killen et al., 2015). While in trapping, social behavior and group cohesion seem to be the more significant factors affecting capture and may modulate the effects of an individual's metabolism. Boldness and activity can be positively correlated with metabolic traits at the individual level, at least in some contexts (Metcalfe et al., 2015). In groups such as fish shoals, however, behaviors among animals tend to become more homogenous and so links between foraging behaviors—including those that relate to trap encounter and engagement—and physiological traits may break down at the individual level. In the current study, the effect of group size appeared to have overwhelmed any effect of metabolic traits. Nevertheless, given that metabolic rates have been found to be correlated to the willingness of fish to shoal (Killen, Fu, et al., 2016), it remains plausible that metabolic phenotypes could be under correlated selection in response to trap fisheries.

A range of additional factors will also play a role in larger scale fisheries and could be examined in the field or with additional laboratory studies. Foraging activity and risk‐taking tendency, for instance, can be labile and highly dependent on context (Killen, Marras, Metcalfe, McKenzie, & Domenici, 2013). Therefore, the alteration of factors such as food availability, temperature, and predation risk could modulate links between capture vulnerability and metabolic phenotype. Trap design and operation, such as the type of bait used, entry size, the choice of habitat for deployment, and deployment duration, could also be crucial in determining the degree to which traps preferentially select for particular phenotypes. In particular, trap deployment time may have a significant influence on the number of fish captured or the phenotypic composition of the final catch (Bacheler, Bartolino, & Reichert, 2013). Future work could examine whether longer deployment times could result in a higher capture rate for those fish that are less exploratory or social, or if this is counterbalanced by increased opportunity for escape among particular phenotypes that entered the trap earlier.

### 4.3 Caveats and considerations

The current study used small‐scale fishing simulations to understand the effects of group size and individual metabolic traits on vulnerability to capture. There is a question of how the results here may be used to understand fisheries in wild fish populations at larger spatial and temporal scales, but the overall strategy in these cases is to use results from simulations to refine lines of inquiry at larger scales and to inform the design of challenging field studies in more complex environments. Simulations allow for manipulation of environmental variables far beyond that which is achievable in a field setting. A limitation in our experiment is that both individual fish and shoals were tested in the same arena, meaning that fish density across trials was not constant. It is, therefore, possible that animal density may have also influenced trap discovery rate. This question could be addressed in a future study by dynamically increasing arena size with group size. Finally, while we consider zebrafish to be a reasonable surrogate for gregarious benthopelagic species (e.g., gadoids), which are often targeted by commercial fisheries, there may be species‐specific differences in behavior or physiology with effects on trap vulnerability. However, the current results indicate that for social fish species, group behavior will strongly affect individual vulnerability to capture and that this issue is worth investigating further at wider scales and with other fish species.

There are a number of factors that need to be considered when determining how the findings in small scale simulations may extrapolate to wild systems. Firstly, capture of fish by fishing gears should be thought to consist of several stages over several spatial scales (Dyer et al., 2009; Hollins et al., 2018; Rudstam, Magnuson, & Tonn, 1984). At the broadest scale, habitat selection may preclude any capture by fishing gear—that is, fish would never be directly exposed to gears unless they share the same space as fishers (Hollins et al., 2018). However, quantifying the isolated and cumulative selective effects of multiple capture stages is extremely difficult, especially when we currently have little or no knowledge of the social and physiological influences on trap vulnerability. In the current study, we chose to examine what is undoubtedly a critical phase—the point at which a fish decides whether or not to enter an encountered trap. Indeed, recent work with passive gears has demonstrated that while gear encounter is a prerequisite for a fish to be captured, it is the decision of whether to interact with the gear after the encounter, that is, actually more important in determining individual vulnerability (Monk & Arlinghaus, 2017). The arena in the current study is roughly equivalent to studying 40 cm gadoids (total length) in a system with 174 × 10^3^ liters of water. Our design focused on the postencounter phase of capture while still accommodating some variation in gear discovery time within the arena. This base of knowledge is vital for expanding the scale of empirical studies and interpreting results.

## 5 CONCLUSIONS

In summary, focal fish in larger shoals were consistently found to be vulnerable to capture by trap as compared to those in smaller shoals. There was some evidence of a negative link between SMR and vulnerability to capture, although group size appeared to overwhelm modulation from metabolic phenotype. Additional work is needed to examine the extent to which group size may be altered via under direct or indirect selection on sociability by trap fisheries, potentially generating an evolutionary shift toward less social fish. The present study contributes to the growing body of evidence suggesting that both behavior and physiology are important aspects FIE (Alós et al., 2016; Biro & Sampson, 2015; Philipp et al., 2009). More information is still needed to answer whether, and to what level, the harvest of fish in the wild can lead to persistent behavioral and physiological change over generations.

## CONFLICT OF INTEREST

None declared.

## AUTHOR CONTRIBUTIONS

DT designed the study, executed the experiments, collected and organized data, and produced a draft of the manuscript. JH and TVL participated in experiments as well as providing some input to various drafts of the manuscript. SK, JL, and KP contributed to the design of the study and contributed to the final drafts of the study. AR was involved with fish husbandry and provided comment on various drafts. All authors read and approved the final manuscript.
